# Minimum-Time Trajectory Generation for Wheeled Mobile Systems Using Bézier Curves with Constraints on Velocity, Acceleration and Jerk

**DOI:** 10.3390/s23041982

**Published:** 2023-02-10

**Authors:** Martina Benko Loknar, Gregor Klančar, Sašo Blažič

**Affiliations:** Faculty of Electrical Engineering, University of Ljubljana, Tržaška cesta 25, SI-1000 Ljubljana, Slovenia

**Keywords:** wheeled mobile robots, trajectory generation, velocity profile, trajectory optimization, Bézier curves

## Abstract

This paper considers the problem of minimum-time smooth trajectory planning for wheeled mobile robots. The smooth path is defined by several Bézier curves and the calculated velocity profiles on individual segments are minimum-time with continuous velocity and acceleration in the joints. We describe a novel solution for the construction of a 5th order Bézier curve that enables a simple and intuitive parameterization. The proposed trajectory optimization considers environment space constraints and constraints on the velocity, acceleration, and jerk. The operation of the trajectory planning algorithm has been demonstrated in two simulations: on a racetrack and in a warehouse environment. Therefore, we have shown that the proposed path construction and trajectory generation algorithm can be applied to a constrained environment and can also be used in real-world driving scenarios.

## 1. Introduction

Path planning and trajectory planning are fundamental topics in autonomous mobile robotics and even more broadly in the context of automation [1]. Path planning algorithms generate a path through predefined points with the main goal of finding a continuous path that minimizes the distance between the starting point and an end point without colliding with obstacles [2,3]. While path planning is a geometric problem, trajectory planning additionally parameterizes the resulting path by time. Consequently, defining time moments along a path affects the kinematic and dynamic properties of the motion of a mobile system. Forces and torques depend on acceleration along a trajectory, while vibrations of its mechanical structure are mainly determined by values of jerk, the time derivative of acceleration [4].

The aim of our work was to solve the problem of minimum-time trajectory generation for wheeled mobile systems with constraints on velocity, acceleration, and jerk in a limited planar space without obstacles. The idea we propose is to apply an optimization method to determine the construction parameters of a Bézier curve primitive such that the resulting travel time on a complete smooth path is minimal. The algorithm we use to compute the minimum-time velocity profile is presented in Ref. [5]. It computes the velocity profile on a predefined path under specified constraints on velocity, radial and tangential acceleration, and radial and tangential jerk.

This paper is organized as follows. Section 2 gives a general overview of the related work. Section 3 introduces the research problem and our main objectives, and Section 4 briefly lists the main contributions. The novel methodology of constructing and parameterizing the fifth-order Bézier curves that make up the resulting geometric path is detailed in Section 5.1. In Section 6, we present two applications of our proposed trajectory generation algorithm, namely the computation of the minimum-time trajectory of a wheeled mobile system on a racetrack (Section 6.1) and in a warehouse (Section 6.2). Our conclusions are drawn in the last section.

## 2. Related Work

The problem of minimum-time trajectory planning remains relevant due to the growing demands for optimal operation of mobile systems, robots, and automated machines. Trajectory planning or, more generally, the planning of the motion of mobile systems can be divided into two parts: velocity profile optimization and path search [6].

The problem of velocity profile optimization is to determine the time-optimal speed law that satisfies certain kinematic or dynamic constraints, and was considered in Refs. [7,8]. The authors in Ref. [9] have provided a comprehensive review of the consideration of jerk in science and engineering, where it is used as a design factor to ensure ride comfort (e.g., amusement park rides, watercraft, elevators, and autonomous buses), and also reference jerk-related ISO standards. As a result, jerk has established its relevance in numerous scientific and engineering applications. Much of the research is dedicated to limiting or minimizing jerk to reduce vibration, decrease positional errors, or improve the overall performance of machine tools [10], robotic manipulators [11,12,13,14,15], and autonomous mobile robots [5,16,17,18,19].

Numerous path planning strategies have been designed and implemented in the literature [20,21,22]. To meet the kinematic limits of the vehicle and successfully transport a hazardous, fragile, or valuable load, the resulting path must be smooth [23]; it must be feasible at high speeds while being harmless to the mechanical system by avoiding vibration and excessive acceleration of the actuators. Often, path planning techniques must also comply with geometric constraints [3,24]. A significant part of path planning methods is the choice of geometric curves, which can be polynomials [25], Bézier curves [6,26,27,28], cubic splines [29], B-splines [30], Dubins curves, clothoids [31], hypocycloids [32], and others, as presented in Ref. [23].

In this work, we have utilized Bézier curves due to their favorable properties, including low computational cost and flexibility. The authors in Refs. [33,34,35,36] also used quintic Bézier curves and various optimization approaches in an attempt to improve the efficiency and accuracy of path planning for autonomous vehicles. In Ref. [33], the author described the cubic and quintic (trigonometric) Bézier curves using a few shape parameters, which makes the method flexible for use in cluttered environments. However, the author only evaluated and compared the values of velocity, radial acceleration, longitudinal and radial jerks on given unit speed curves. In Ref. [34], the authors proposed a real-time motion planning approach for automated driving in urban environments. Similar to our case, they used a decoupled method by separating path and speed planning. While their trajectory generation approach is suitable for environments with obstacles, the generated velocity profiles do not include jerk constraints. In Ref. [35], the presented method combines jump point search with Bézier curves. However, their approach only ensures $C^{2}$ continuity and considers velocity and acceleration constraints. In Ref. [36], the authors proposed an optimization approach for path planning for driverless vehicles in parallel parking using a radial basis function neural network. The authors optimized performance to ensure curve continuity, safety, and compliance with curvature constraints, but did not address the problem of velocity planning or compliance with other dynamic constraints.

Mobile robots are finding broader application and have become an integral part of a variety of environments: in manufacturing, medicine, and many other robotics-based services, including automated warehouses [37,38,39,40]. In work environments where simple and labor-intensive tasks of workers are replaced by mobile robots, labor efficiency, scalability, adaptability, and warehouse visibility increase, and turnaround time decreases.

## 3. Problem Formulation

Let the motion of a mobile system along a three times continuously differentiable plane curve C be described as a function of time $t\in \left[0,t_{f}\right]$ by the position vector r(t) measured from a given fixed origin. The velocity vector v(t), the acceleration vector a(t), and the jerk vector j(t) in the tangential-normal form are:

(1a)v(t)=v(t)·T^,(1b)a(t)=aT(t)·T^+aR(t)·N^=v˙·T^+v2κ·N^,(1c)j(t)=jT(t)·T^+jR(t)·N^=v¨−v3κ2·T^+1vddt(v3κ)·N^,

where $\hat{T}$ and $\hat{N}$ are the unit tangential and the unit normal vector, respectively, and κ is the curvature of the path at time *t*. In Equation (1a), *v* is called speed, and the tangential and normal components of the acceleration (Equation (1b)) are called acceleration along the path and centripetal acceleration (also called radial acceleration), respectively. The expression (1c) is obtained by differentiating Equation (1b) and applying the Frenet–Serret formulas for movement in two-dimensional Euclidean space $R^{2}$ [41].

The maximum allowable value of velocity $v_{\mathrm{MAX}}$ is determined by the capabilities of the robot actuators and also the environmental conditions (e.g., surface type). Driving in the reverse direction is not permitted. The maximum values of radial acceleration $a_{R_{\mathrm{MAX}}}$ and tangential acceleration $a_{T_{\mathrm{MAX}}}$ can be set based on the dynamic constraints of a mobile robot (e.g., maximum centripetal force and rolling resistance in a turn) [6]. Similarly, we imposed third-order constraints $j_{R_{\mathrm{MAX}}}$ and $j_{T_{\mathrm{MAX}}}$, whose values can be derived from ride comfort criteria [9]. Although we could limit the acceleration and jerk components separately (and treat them individually), we additionally restricted their values to the range within an ellipse (similar to researchers in [5,6,35,42]:

(2a)0≤|v(t)|≤vMAX,(2b)aR2(t)aRMAX2+aT2(t)aTMAX2≤1,(2c)jR2(t)jRMAX2+jT2(t)jTMAX2≤1.

Treating the tangential and radial components of acceleration (Equation (2b)) and jerk (Equation (2c)) together is more rigorous than limiting the individual components. It also provides greater ride comfort by constraining the overall norms. The goal of this research was to develop a trajectory planning method for a mobile system operating in a constrained, obstacle-free, planar environment while subject to kinematic constraints. Although motion planning algorithms have been the subject of extensive research, dealing with third-order constraints still proves challenging.

## 4. Contributions

The main contributions of this paper can be summarized as follows:

- We describe an innovative construction method for 5th order Bézier curves. The proposed parameterization is simple and intuitive, yet effective for generating smooth paths consisting of multiple splines (Section 5);
- The above smooth path generation basis is coupled with an algorithm that computes a minimum-time velocity profile with velocity, acceleration, and jerk constraints on a predefined path (see Ref. [5]). Together they form a powerful trajectory generation algorithm (Section 6). The resulting trajectories thus provide continuous velocity and acceleration profiles;
- To prove the applicability of our approach to trajectory optimization, we performed simulation experiments on a racetrack and in a warehouse environment (Section 6.1 and Section 6.2). In the warehouse simulation, we identified and analyzed realistic situations with different dynamic constraints to investigate and propose the most appropriate driving scenarios.

## 5. Curve Primitives

A Bernstein–Bézier curve (or Bézier curve) is defined by a set of control points $P_{0},P_{1},\cdots P_{b}$, b∈N:

(3) $$r_{b}\left(\lambda \right)=\sum_{i=0}^{b}p_{i}B_{i,b}\left(\lambda \right),$$

where λ is a normalized time variable (0≤λ≤1) and $p_{i}$ denotes the position vector of a control point $P_{i}$. The polynomials $B_{i,b}\left(\lambda \right)$:

(4) Bi,b(λ)=biλi(1−λ)b−i=b!i!(b−i)!λi(1−λ)b−i,

are known as Bernstein basis polynomials of degree *b*. Bézier curves can be defined for *N*-dimensional space, N∈N. In planar space, the curve $r_{b}\left(\lambda \right)$ and the vectors $p_{i}$ are two-element vectors: $r_{b}\left(\lambda \right)={\left[X(\lambda ),Y(\lambda )\right]}^{T}$ and $p_{i}={\left[x_{i},y_{i}\right]}^{T}$. These curves have several useful properties for path planning. The first and last points of the Bézier curves introduced in Equation (3) are their endpoints:

(5) $$r_{b}\left(0\right)=p_{0}\;\mathrm{and}\;r_{b}\left(1\right)=p_{b}.$$

**Figure 1:**
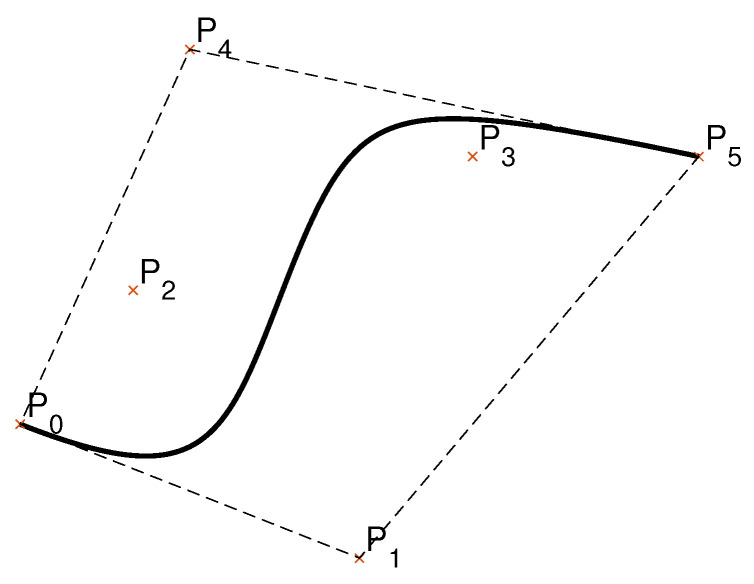
Fifth order Bernstein–Bézier curve within its convex hull (dashed lines). The curve is tangent to the sides of the convex hull, line segments $\bar{P_{0}P_{1}}$ and $\bar{P_{4}P_{5}}$.

The *N*-dimensional, *b*-th order Bézier curve also lies within the convex hull defined by its control points. Furthermore, the beginning and the end of the curve are tangent to the first and the last section of the convex polygon, respectively (Figure 1).

(6) $$\begin{array}{cc} {\left.\frac{dr_{b}}{d\lambda }\right|}_{\lambda =0} & =b(p_{1}-p_{0}), \end{array}$$

(7) $$\begin{array}{cc} {\left.\frac{dr_{b}}{d\lambda }\right|}_{\lambda =1} & =b(p_{b}-p_{b-1}). \end{array}$$

Other properties of Bernstein polynomials (derivatives, calculation of definite integrals, the de Casteljau algorithm, degree elevation, etc.) fall outside the scope of this article; more details on this topic can be found in Ref. [28].

Bézier curves constructed by a large number of control points are computationally intensive. For this reason, in path planning, it is desirable to construct a smooth path by connecting low-degree Bézier curves [6]. The authors in Ref. [43] proposed a new parameterization of motion primitives based on Bézier curves for path planning applications of wheeled mobile robots. However, the method was presented for third-order polynomials and the algorithm does not guarantee the existence of the curve for all possible parameterizations. We used fifth-order Bézier curves because this is the degree of Bézier curves that always satisfies the curvature continuity requirement ($C^{2}$) in the joints. The 5th order Bézier curve $r_{5}\left(\lambda \right)$ is defined by six control points $P_{i}$:

r5(λ)=(1−λ)5p0+5λ(1−λ)4p1+10λ2(1−λ)3p2+10λ3(1−λ)2p3(8)+5λ4(1−λ)p4+λ5p5.

### 5.1. Construction of 5th Order Bézier Curves

It is very important to choose the appropriate construction parameters that would efficiently define the Bézier curves and facilitate the search for the minimum-time trajectory.

**Figure 2:**
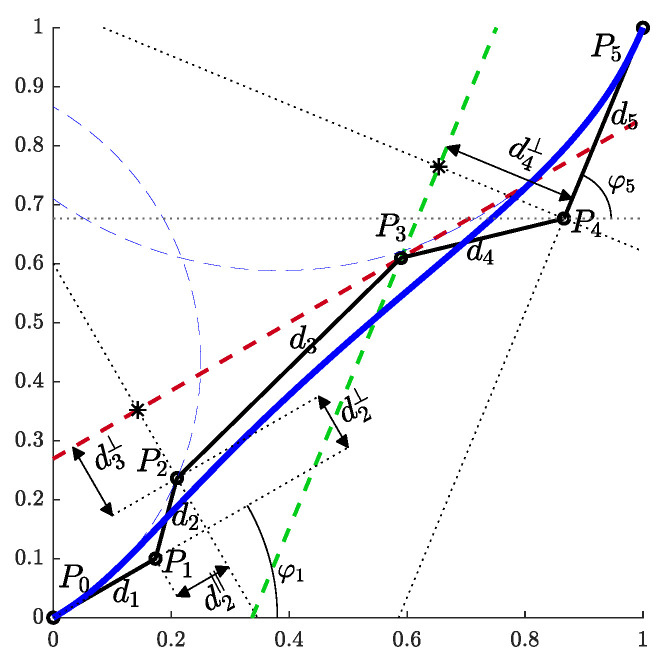
The proposed construction of a Bézier curve that enables efficient parameterization.

With the above notation, let us mark the distances between consecutive control points $d(P_{i},P_{i+1})$ as $d_{i+1}$ and the angles between ($P_{i},P_{i+1}$) and the positive direction of the *x*-axis as $\varphi_{i+1}$ (Figure 2), $i=\left\{0,1\cdots ,4\right\}$. For the coordinates of two consecutive control points, it follows that:

(9a) $$\begin{array}{c} x_{i+1}-x_{i}=d_{i+1}\cos\varphi_{i+1}, \end{array}$$

(9b) $$\begin{array}{c} y_{i+1}-y_{i}=d_{i+1}\sin\varphi_{i+1}. \end{array}$$

We evaluate (9a) and (9b) for i∈{0,1} using the sum and difference formulas for sine and cosine. This gives the following expression for the value of the curvature in $P_{0}$:

(10) $$\begin{array}{c} \lim_{\lambda \to 0}\kappa \left(\lambda \right)=\kappa_{0}=\frac{4}{5}\frac{d_{2}}{d_{1}^{2}}\sin\left(\varphi_{2}-\varphi_{1}\right). \end{array}$$

The derivative of curvature $\kappa_{0}$ in $P_{0}$ with respect to λ is:

(11) $$\lim_{\lambda \to 0}\frac{d\kappa }{d\lambda }=\frac{12}{5}\frac{1}{d_{1}^{2}}d_{3}\sin\left(\varphi_{3}-\varphi_{1}\right)+\kappa_{0}\left(-12\frac{d_{2}}{d_{1}}\cos\left(\varphi_{2}-\varphi_{1}\right)+6\right).$$

We choose the parameters of the curve so that the second term in Equation (11) becomes 0. This happens when:

(12) $$\begin{array}{c} \frac{d_{2}}{d_{1}}=\frac{1}{2\cos(\varphi_{2}-\varphi_{1})}. \end{array}$$

The curvature $\kappa_{0}$ from Equation (10) and its derivative $\kappa_{0}^{\prime }$ in $P_{0}$ from Equation (11) then become:

(13a)κ0=410tan(φ2−φ1)d1,(13b)κ0′=125d3sin(φ3−φ1)d12.

The purpose of introducing notations for $d_{i}$ and $\varphi_{i}$ and deriving expressions for $\kappa_{0}$ and $\kappa_{0}^{\prime }$ is to make the process of path construction as efficient and intuitive as possible. This also takes into account that the path ultimately consists of several Bézier curves. Thus, the parameters needed to generate a 5th order Bézier curve are $P_{0},P_{5},\varphi_{1},\varphi_{5},\kappa_{0},\kappa_{5},d_{1},d_{5}$, and $\kappa_{0}^{\prime }$. However, how would one set the value of $\kappa_{0}^{\prime }$? It could be simply set to zero, but perhaps it is also useful to examine Equation (1c) and choose such a value for $\kappa_{0}^{\prime }$ that the value of the radial component (in $P_{0}$) of the jerk vector is zero.

A 5th order Bézier curve is therefore constructed in the following steps (Figure 2):

(1) Outline the first control point and mark it as $P_{0}$. In the direction of $\varphi_{1}$, measure out the distance $d_{1}$ and mark the second point as $P_{1}$.
(2) In the direction $\varphi_{1}$, measure out the distance $d_{2}^{\left|\right|}$ (from Equation (12)):
  (14) $$\begin{array}{c} d_{2}^{\left|\right|}=d_{2}\cos\left(\varphi_{2}-\varphi_{1}\right)=\frac{1}{2}d_{1}. \end{array}$$
(3) Measure in the perpendicular direction the distance $d_{2}^{⊥}$ (from Equation (10)):
  (15) $$\begin{array}{c} d_{2}^{⊥}=\frac{5}{4}d_{1}^{2}\kappa_{0}. \end{array}$$
  and mark the third point as $P_{2}$.
(4) All points away from $P_{2}$ for $d_{3}^{⊥}$ (Equation (11)) in the same direction (perpendicular to the line segment $\bar{P_{0}P_{1}}$) lie on the red dashed line.
  (16) $$\begin{array}{c} d_{3}^{⊥}=\frac{5}{12}d_{1}^{2}\kappa_{0}^{\prime }. \end{array}$$
(5) Mark the last point as $P_{5}$. Measure out the distance $d_{5}$ in the opposite direction from $\varphi_{5}$ and mark the fifth point as $P_{4}$.
(6) All points away from $P_{4}$ for $d_{4}^{⊥}$ (Equations (9a), (9b) and (10) for i=4) in the same direction (perpendicular to the line segment $\bar{P_{4}P_{5}}$) lie on the green dashed line:
  (17) $$\begin{array}{c} d_{4}^{⊥}=\frac{5}{4}d_{5}^{2}\kappa_{5}. \end{array}$$
(7) The fourth control point $P_{3}$ lies on the intersection of the red and green dashed lines. The Bézier curve is now completely defined.

## 6. Generation of Minimum-Time Trajectories

We have shown the use of the proposed trajectory planning algorithm in two environments. On a racetrack, the focus was on demonstrating path construction and ensuring that it is within the corridor boundaries. In a warehouse, we demonstrated the benefits of our proposed methods in a real-world application. All simulations were performed using the Matlab programming environment on a computer with an Intel(R) Core(TM) i7-8700 CPU 3.2 GHz processor with 16 GB RAM memory.

A minimum-time trajectory is computed by applying an algorithm that generates a minimum-time velocity profile (proposed in Ref. [5]) to Bézier curve splines. The algorithm, which considers velocity, acceleration, and jerk constraints along a given path, consists of two steps. In the first step of the algorithm, the velocity and acceleration constraints are considered. In the second step, the algorithm modifies the original velocity profile to include the jerk constraints, with the process varying depending on the type of violation (single-point or interval jerk violations). The simulation methodology for computing the minimum-time velocity profile, described in detail in Ref. [5], can essentially be described as solving the presented ordinary differential equation with a given initial value. In our own implementation, numerical methods (Euler’s method and trapezoidal integration) were used to calculate the required values in the discrete time samples.

We then used a nonlinear gradient optimization method, an optimization routine built into Matlab, to change the construction parameters of the Bézier curves. Using this method, we found a solution where the travel time reached a minimum. Since the simulated environments were static and free of obstacles, we divided the environments into several individual sections. This was done primarily to reduce the number of optimization parameters and consequently speed up the minimization process.

### 6.1. Racetrack Environment

**Figure 3:**
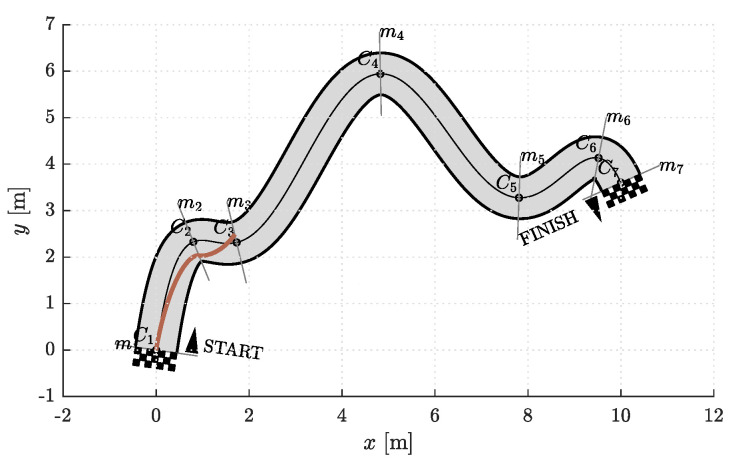
The racetrack model we used for the simulations. Shown are points $C_{i}$ on the centerline where the curvature is locally highest, and lines $m_{i}$ where $P_{1}^{i}$ and $P_{5}^{i}$ lie.

The model of a racetrack that we used in our simulations is shown in Figure 3. It is defined by the centerline. The left and right edges of the racetrack are at a distance w/2 from the centerline and represent the corridor boundaries. The shape of the racetrack can in general be arbitrary complex and is therefore divided into segments. This is done by analyzing the curvature of the centerline. Points where the curvature reaches local extrema are denoted by the sequence $C_{i}$, $i\in \{1,\cdots ,N_{\mathrm{parts}}+1\}$ where $N_{\mathrm{parts}}$ is the number of segments. Then perpendicular lines to the centerline are drawn in $C_{i}$. These lines represent the edges of individual segments. The first and last control points of the Bézier curves lie somewhere on the segment edges. For simplicity, we represent the positions of $P_{0}^{i}$ and $P_{5}^{i}$, $i\in \{1,\cdots ,N_{\mathrm{parts}}\}$, by the parameter $p\in \left[-1,1\right]$. The sign of *p* indicates whether the control point lies somewhere between the centerline and the right (+) or left (−) edge of the racetrack.

**Figure 4:**
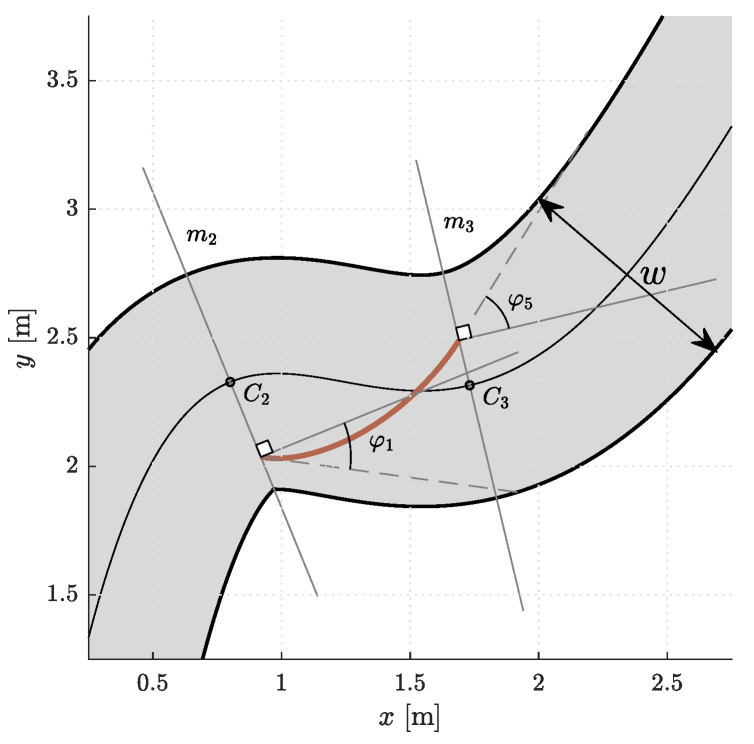
A segment with a Bézier curve. $\varphi_{1}$ and $\varphi_{5}$ are measured from the line perpendicular to $m_{i}$.

**Figure 5:**
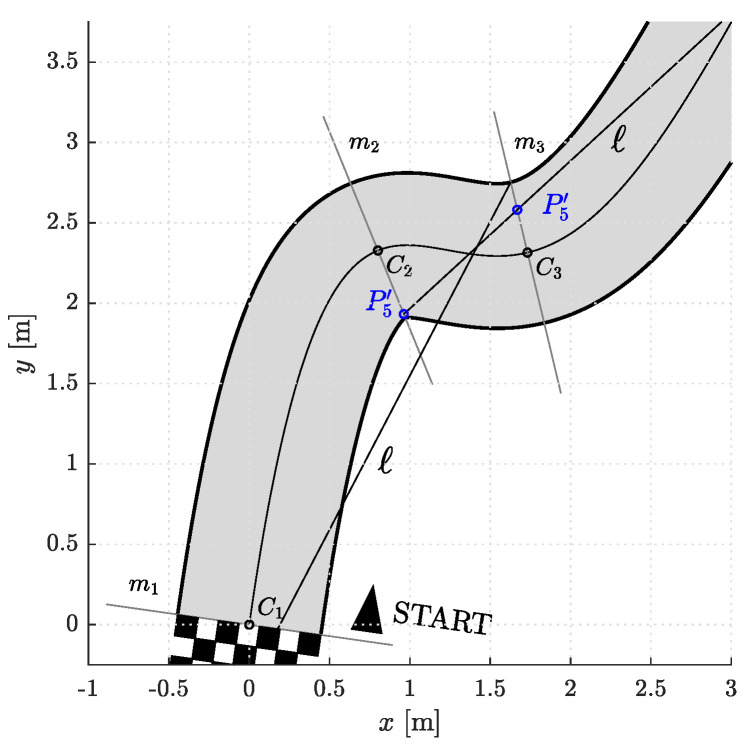
An example of heuristic determination of initial guesses for construction of Bézier curve(s).

Thus, each curve in a segment is completely defined by the positions, angles, and curvatures in the first and last control points ($p_{0}$, $\varphi_{1}$, $\kappa_{0}$, $p_{5}$, $\varphi_{5}$, $\kappa_{5}$), $d_{1}$, and $d_{5}$. Note that the angles are measured from a tangent to the centerline in $C_{i}$ (Figure 4). To find a reasonable starting point for the optimization, we devised a simple heuristics. In each segment, a line *ℓ* is drawn from $P_{0}^{i}$ through the outermost edge of the inner side of the corridor at the end of the (i+1)th segment. The intersection of lines *ℓ* and $m_{i}$ is the initial estimate for the position $p_{5,\mathrm{init}}^{i}$ of the last control point $P_{5}^{\prime i}$. If the intersection point is outside the corridor (as in Figure 5), $p_{5,\mathrm{init}}^{i}$ is set to its edge. The initial estimate for the angle $\varphi_{5,\mathrm{init}}^{i}$ is the angle between the line *ℓ* and the line perpendicular to $m_{i+1}$, while $\kappa_{5,\mathrm{init}}^{i}$ is the curvature of the centerline in $C_{i+1}$. The values of $d_{1}^{i}$ and $d_{5}^{i}$ were set to the value of a certain fraction of the distance between $P_{0}^{i}$ and $P_{5}^{\prime i}$. The heuristic procedure described is shown in Figure 5. To simplify the notation, we will from now on omit the superscript *i*.

We devised a series of separate simulation experiments to demonstrate the operation and efficiency of the proposed Bézier curve construction and trajectory planning algorithm.

Let $N_{\mathrm{free}}$ denote the number of optimization parameters on each corridor segment. It is expected that the higher the number of (free) curve construction parameters $N_{\mathrm{free}}$, the more versatile a curve is. Thus a better solution can be provided. However, in this way the optimization problem becomes more computationally expensive and the solution more difficult to obtain due to the complex form of the objective function.

Another problem is the gradual generation of the final trajectory. A Bézier curve can be constructed for each segment separately and have the criterion assigned to it. Alternatively, multiple Bézier curves spanning several segments can be constructed together, and the objective is the travel time along all of them. Then only the solution on the first segment is kept and this procedure is repeated in a receding horizon manner. Let us denote by $N_{\mathrm{seg}}$ the number of segments that are treated simultaneously. If only a one-segment optimization is performed ($N_{\mathrm{seg}}=1$), the solution does not take into account the corridor shape in the following segment, e.g., when a sharp turn follows. On the other extreme, the complete curve (on all corridor segments) can be generated in each run of the optimization, but since the dimension of the optimization problem is the product of the construction parameters, namely $N_{\mathrm{free}}\times N_{\mathrm{seg}}$, it is reasonable not to exaggerate the two values and to find a sensible compromise.

Simulations were performed for $N_{\mathrm{free}}\in \left\{2,5\right\}$ and $N_{\mathrm{seg}}\in \left\{1,2,3\right\}$. Therefore, in one group of experiments, the two optimization parameters are $d_{1}$ and $d_{5}$, while the other three necessary parameters for curve construction ($p_{5}$, $\varphi_{5}$, and $\kappa_{5}$) are given by the heuristics discussed above and shown in Figure 5. In the other set of simulations, there are five optimization parameters, namely $d_{1}$, $d_{5}$, $p_{5}$, $\varphi_{5}$, and $\kappa_{5}$. Certainly, both simulation scenarios also include cases with different $N_{\mathrm{seg}}$. The data obtained from the simulation experiments are compiled in Table 1, where $t_{i}$ represents the travel time at the end of a given corridor segment and $\sum_{i=1}^{6}t_{i}$ is the total travel time on a resulting path.

**Figure 6:**
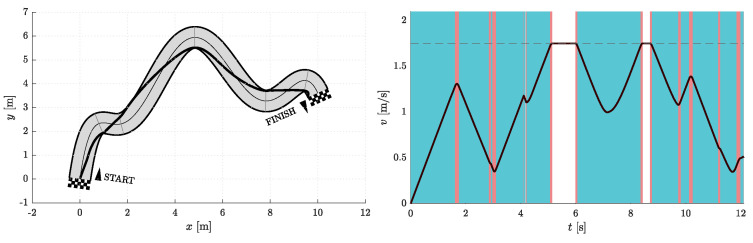
Resulting path as optimization result for $N_{\mathrm{free}}=2$ and $N_{\mathrm{seg}}=1$ (**left**) with the corresponding velocity profile (**right**). Blue vertical bands indicate intervals where the acceleration reaches its maximum allowable values. Similarly, red vertical bands indicate intervals where the jerk reaches its maximum allowable value.

**Figure 7:**
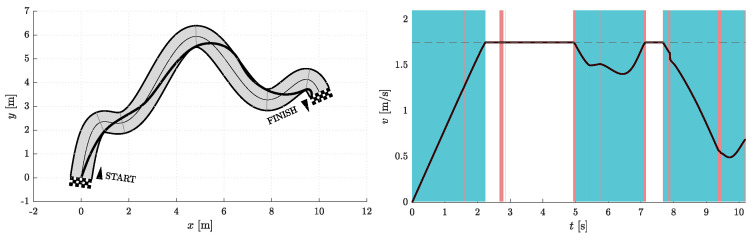
Resulting path as optimization result for $N_{\mathrm{free}}=5$ and $N_{\mathrm{seg}}=1$ (**left**) with the corresponding velocity profile (**right**). Blue vertical bands indicate intervals where the acceleration reaches its maximum allowable values. Similarly, red vertical bands indicate intervals where the jerk reaches its maximum allowable value.

**Figure 8:**
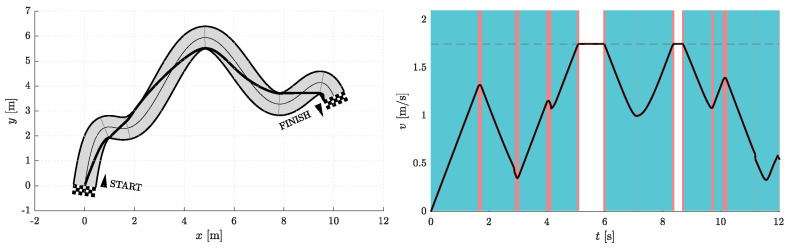
Resulting path as optimization result for $N_{\mathrm{free}}=2$ and $N_{\mathrm{seg}}=2$ (**left**) with the corresponding velocity profile (**right**). Blue vertical bands indicate intervals where the acceleration reaches its maximum allowable values. Similarly, red vertical bands indicate intervals where the jerk reaches its maximum allowable value.

**Figure 9:**
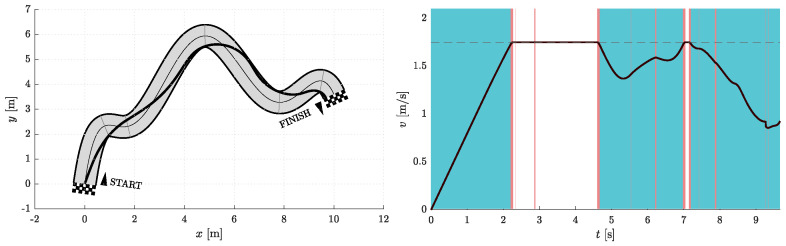
Resulting path as optimization result for $N_{\mathrm{free}}=5$ and $N_{\mathrm{seg}}=2$ (**left**) with corresponding velocity profile (**right**). Blue vertical bands indicate intervals where the acceleration reaches its maximum allowable values. Similarly, red vertical bands indicate intervals where the jerk reaches its maximum allowable value.

**Figure 10:**
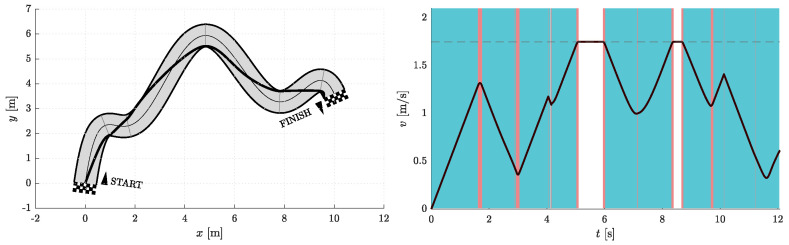
Resulting path as optimization result for $N_{\mathrm{free}}=2$ and $N_{\mathrm{seg}}=3$ (**left**) with the corresponding velocity profile (**right**). Blue vertical bands indicate intervals where the acceleration reaches its maximum allowable values. Similarly, red vertical bands indicate intervals where the jerk reaches its maximum allowable value.

**Figure 11:**
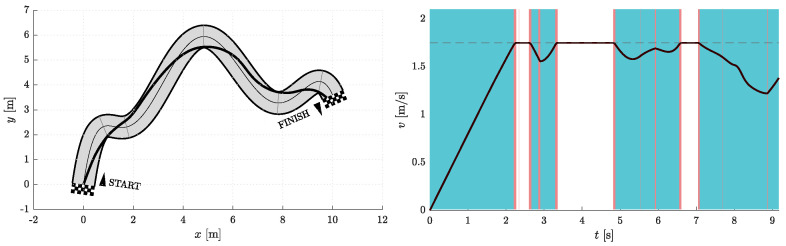
Resulting path as optimization result for $N_{\mathrm{free}}=5$ and $N_{\mathrm{seg}}=3$ (**left**) with the corresponding velocity profile (**right**). Blue vertical bands indicate intervals where the acceleration reaches its maximum allowable values. Similarly, red vertical bands indicate intervals where the jerk reaches its maximum allowable value.

Figure 6, Figure 7, Figure 8, Figure 9, Figure 10 and Figure 11 show the resulting paths in the racetrack and the corresponding velocity profiles. We imposed the following constraints: $v_{\mathrm{MAX}}=$1.75 m/s (represented by the dashed horizontal lines), $a_{R_{\mathrm{MAX}}}$ = 1.6 m/s${}^{2}$, $a_{T_{\mathrm{MAX}}}$ = 0.8 m/s${}^{2}$, $j_{R_{\mathrm{MAX}}}$ = 16 m/s${}^{3}$, $j_{T_{\mathrm{MAX}}}$ = 12 m/s${}^{3}$.

As expected, the results in Table 1 show that the travel times decrease when either $N_{\mathrm{seg}}$ or $N_{\mathrm{free}}$ increases. The shortest travel time is calculated for the case where $N_{\mathrm{seg}}$ = 3 and $N_{\mathrm{free}}$ = 5.

### 6.2. Warehouse Environment

The enormous technological capabilities of automated guided vehicles (AGVs) and other autonomous mobile robots (AMRs) are facilitating the launch of fully automated warehouses. Common warehouse tasks performed by mobile robots include loading and unloading goods, stacking and retrieving items, picking and sorting orders, inventory tracking, and warehouse maintenance.

**Figure 12:**
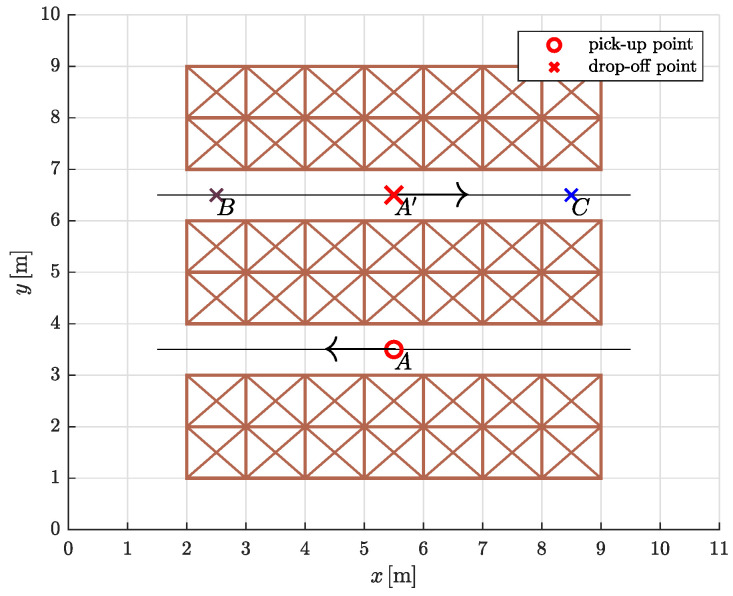
The warehouse floor plan with three pairs of pick-up and drop-off points: *A* and $A^{\prime }$, *A* and *B*, *A* and *C*.

**Figure 13:**
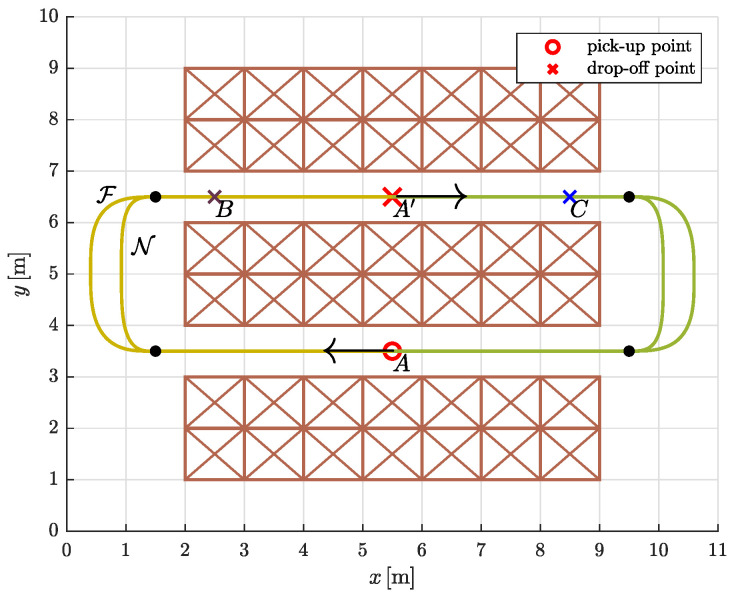
The drawn paths F and N are the result of an optimization that minimizes travel time. The filled dots mark the points where the straight segments meet the Bézier curves.

We tested the proposed trajectory planning algorithm in a simple warehouse environment by simulating the task of moving between three rows of storage racks, picking up and dropping off loads from specific locations (Figure 12). Warehouses are usually very confined environments, so we assumed that movement in the two aisles is restricted to a straight line. To avoid collisions of AGVs with storage racks, the straight segments on both sides protrude slightly beyond the edges (black solid dots in Figure 13). The optimization problem is to find the most suitable path shape between the aisles.

The simulation experiment was designed as follows. An AGV travels clockwise along a circular route from the **p**ick-**u**p **p**oint (PUP) to the **d**rop-**o**ff **p**oint (DOP) and back to the starting point. At the pick-up and drop-off points, the speed is set to zero. As the load is delicate, the dynamic constraints on the mobile system are more severe in the first part of the path, as shown in Table 2.

We proposed that the continuous curvature path between a pick-up point and a drop-off point (and vice versa) consists of two straight lines and two 5th order Bézier curves. The coordinates of the control points were determined by an optimization process that minimizes travel time. Since the velocity is set to zero at the symmetrically placed drop-off point $A^{\prime }$, the optimizations can be performed only on one half of the circular route (thus on four segments instead of eight). The free optimization parameters for the construction of the Bézier curves were $d_{1}$ and $d_{5}$ (Section 5.1 for a full explanation) and the *x* coordinate of the joint between them, which is $P_{5}$ of the first Bézier curve and $P_{0}$ of the second Bézier curve.

First, we computed the minimum-time trajectories for symmetrically placed pair of pick-up and drop-off point (*A* and $A^{\prime }$). Let us denote the path representing the **f**ull-load optimization solution for the symmetrically placed pair *A* and $A^{\prime }$ by F. Similarly, let N be the path representing the **n**o-load optimization solution for the symmetrically placed pair *A* and $A^{\prime }$ (Figure 13).

We then conducted a comparative travel time analysis. The main question was whether travel times differ in cases where a fully loaded/unloaded AGV travels along a path that is not optimized for a load of the same type. Normally, AGVs in warehouses travel along predefined trajectories. So with the simulation experiment, we wanted to test whether it is possible to reduce travel time if each curve segment is optimized for the actual load being carried. We also included examples with different ratios of travel times (or path lengths) of fully loaded or unloaded mobile systems by adding drop points *B* and *C*. Essentially, we calculated travel times for the three pick-up and drop-off pairs where the AGV was fully loaded on the first part (PUP→DOP) and unloaded on the second part (DOP→PUP) of the circular path, but traveling on either F or N. The travel times are given in Table 3, where the subscripts indicate the load type of the AGV. By μ, we denote the relative increase (in percent) in travel time in a given route case scenario compared to the travel time when the AGV travels on a path optimized for a load of the same type (the last three rows in Table 3).

The results in Table 3 show that the travel time is indeed the shortest when the mobile system travels along the route optimized for the actual load (PUP→DOP: $F_{F}$, and DOP→PUP: $N_{N}$). Moreover, it can be seen that when the default path is F (rows 4–6 in Table 3), the corresponding travel times are always shorter than in the case when the default path is N (rows 1–3 in Table 3). However, generally, the travel times are not radically different and this observation is not entirely unexpected. We could achieve more obvious travel time differences if we increased the ratio of fully loaded to unloaded constraint values (see Table 2) or chose a more complex arrangement of pick-up and drop-off points spanning multiple rows of storage racks. Nevertheless, the selected values for velocity, acceleration, and jerk constraints (and the ratio between the two load types) should reflect reality. Additionally, the examples presented can be viewed as the smallest units for which this analysis can be performed. These subtle differences in travel times (approximately 1% reduction) imply significant absolute time differences when the presented trajectories are combined into larger trajectories. Or if one considers that a warehouse robot would traverse the same trajectories over and over again during its entire operation.

**Figure 14:**
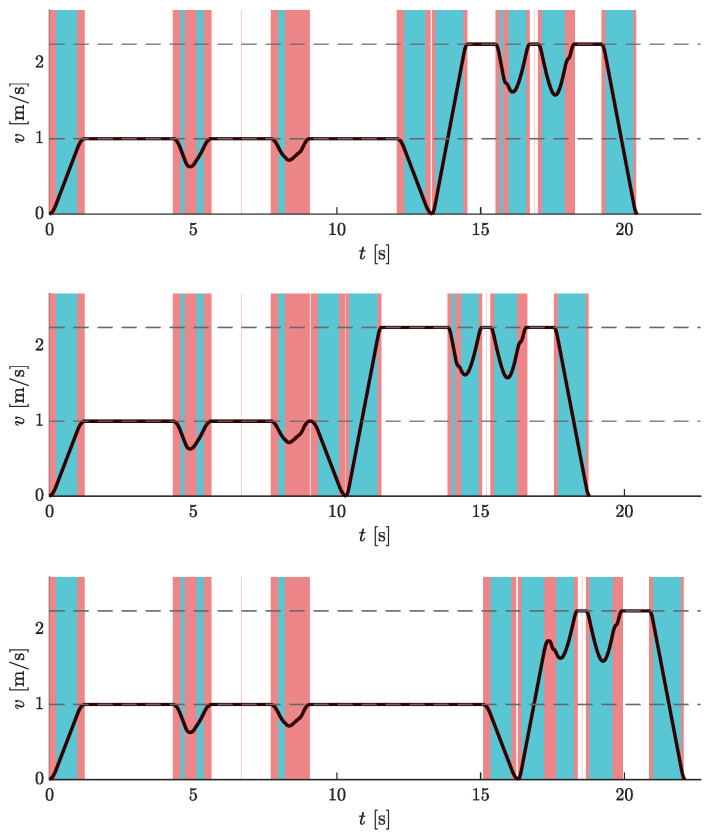
Velocity profiles of AGV traveling on a circular route for different placements of pick-up and drop-off points: *A* and $A^{\prime }$ (**top**), *A* and *B* (**middle**), *A* and *C* (**bottom**). The graphs show the results for PUP→DOP: $F_{F}$, and DOP→PUP: $N_{N}$. Dashed horizontal lines represent the velocity limit values. Blue vertical bands indicate intervals where the acceleration reaches its maximum allowable values. Similarly, red vertical bands indicate intervals when the jerk reaches its maximum allowable values.

Figure 14 shows the velocity profiles for all three pairs of pick-up and drop-off points for the case where $F_{F}$ is on the first part and $N_{N}$ is on the second part of the circular route.

## 7. Conclusions

In this paper, we present a new minimum-time optimization-based approach for planning the trajectory of a mobile robot in a planar constrained environment. We assumed that a mobile system has constraints on velocity, acceleration, and jerk. The resulting smooth path consists of 5th order Bézier curves, for whose construction we propose a new method that allows efficient parameterization.

We analyzed the results of the proposed approach for generating trajectories in a simulated automated warehouse. Different sets of dynamic constraints led to different solutions for trajectories. We have shown that it is possible to achieve noticeable improvements in travel times by choosing the appropriate trajectories. The approach is applicable for trajectory and velocity planning of a single wheeled robot, but could be extended for the use of multiple robots to take into account evasive maneuvers or cooperation on a given task. It can also be used by various other mobile systems moving in a plane (e.g., track robots, robotic manipulators), especially non-holonomic systems.

Our findings may be especially useful and have great potential for determining minimum-time trajectories in automated warehouses, where the dynamic constraints imposed on autonomous mobile robots may depend on the type of load the mobile system is transporting. Our approach could also be applied to other planar environments with similar requirements.

The values of the constraints in the warehouse environment were conservatively estimated to ensure the vertical stability of a mobile system. However, the stability of the system (mobile robot with load) itself was not the subject of our research. Future studies should aim to describe the characteristics of the load in more detail, as this could impose additional or more demanding constraints on a mobile system. For a specific mobile system with known load characteristics (mass, mass distribution, dimensions, contact area conditions) it would be possible to calculate the tipping angle and consequently determine the allowable accelerations. The use of higher order Bézier curves or other curve primitives would also be of particular interest. More broadly, research is needed to apply the proposed trajectory planning approach to environments with static or dynamic obstacles to demonstrate the proposed idea using global or local path planning methods.

## Figures and Tables

**Table 1:** Resulting travel times on segments and total travel times. Simulations were performed for different numbers of optimization parameters $N_{\mathrm{free}}\in \left\{2,5\right\}$, and different numbers of segments $N_{\mathrm{seg}}\in \left\{1,2,3\right\}$, whose geometry was taken into account when calculating the solution for the current segment.

$N_{\mathrm{seg}}$	$N_{\mathrm{free}}$	$t_{1}$	$t_{2}$	$t_{3}$	$t_{4}$	$t_{5}$	$\sum_{i=1}^{6}t_{i}$
		[s]	[s]	[s]	[s]	[s]	[s]
1	2	2.91	4.20	7.18	9.73	11.24	12.11
	5	2.34	2.87	5.44	7.89	9.42	10.20
2	2	2.87	4.15	7.14	9.69	11.19	12.03
	5	2.34	2.88	5.54	7.90	9.27	9.66
2	2	2.89	4.16	7.13	9.68	11.22	12.07
	5	2.34	2.90	5.52	7.68	8.87	9.17

**Table 2:** Constraints on velocity, acceleration, and jerk for a fully loaded (✓) and an unloaded (×) mobile system.

Load	$v_{\mathrm{MAX}}$	$a_{R_{\mathrm{MAX}}}$	$a_{T_{\mathrm{MAX}}}$	$j_{R_{\mathrm{MAX}}}$	$j_{T_{\mathrm{MAX}}}$
	[m/s]	[m/s${}^{2}$]	[m/s${}^{2}$]	[m/s${}^{3}$]	[m/s${}^{3}$]
✓	1.0	2.0	1.0	4.0	4.0
×	2.25	4.0	2.0	16	16

**Table 3:** Travel times of the AGV on a circular route for different placements of pick-up (PUP) and drop-off points (DOP). For the symmetrically placed pair $A-A^{\prime }$, F and N denote the paths representing the optimization solutions **f**ull-load and **n**o-load, respectively. Similarly, the indices F and N denote the type of load on the AGV. We write μ for the increase in travel time according to the last three rows, expressed as a percentage.

Circular Route Case		Pick Up Point	Drop off Point	Travel Time	μ
PUP → DOP	DOP → PUP	(PUP)	(DOP)	[s]	**[%]**
$N_{F}$	$N_{N}$	*A*	$A^{\prime }$	20.74	1.49
		*A*	*B*	19.08	1.62
		*A*	*C*	22.40	1.38
$F_{F}$	$F_{N}$	*A*	$A^{\prime }$	20.65	1.04
		*A*	*B*	18.99	1.13
		*A*	*C*	22.25	0.66
$F_{F}$	$N_{N}$	*A*	$A^{\prime }$	20.44	0
		*A*	*B*	18.78	0
		*A*	*C*	22.10	0

## Data Availability

Not applicable.
